# A Hybrid Multi-Scale Transformer-CNN UNet for Crowd Counting

**DOI:** 10.3390/s26010333

**Published:** 2026-01-04

**Authors:** Kai Zhao, Chunhao He, Shufan Peng, Tianliang Lu

**Affiliations:** 1 School of Information Network Security, People’s Public Security University of China, Beijing 100038, China; 2023112042@stu.ppsuc.edu.cn (K.Z.);; 2 School of Cyber Security and Smart Policing, Zhengzhou Police University, Zhengzhou 450000, China; 3 Research Center for Cybersecurity and Artificial Intelligence, People’s Public Security University of China, Beijing 100038, China

**Keywords:** crowd counting, HMSTUNet, vision transformer, multi-scale learning, UNet

## Abstract

Crowd counting is a critical computer vision task with significant applications in public security and smart city systems. While deep learning has markedly improved accuracy, persistent challenges include extreme scale variations, severe occlusion, and complex background clutter. To address these issues, we propose a novel Hybrid Multi-Scale Transformer-CNN U-shaped Network (HMSTUNet). Our key contributions are: a hybrid architecture integrating a Multi-Scale Vision Transformer (MSViT) for capturing long-range dependencies and a Dynamic Convolutional Attention Block (DCAB) for modeling local density patterns; and a U-shaped encoder–decoder with skip connections for effective multi-level feature fusion. Extensive evaluations on five public benchmarks show that HMSTUNet achieves the best Mean Absolute Error (MAE) on all five datasets and the best Mean Squared Error (MSE) on three. It sets new state-of-the-art records, attaining MAE/MSE of 49.1/77.8 on SHA, 6.2/10.3 on SHB, 142.1/192.7 on UCF_CC_50, 77.9/132.5 on UCF-QNRF, and 43.2/119.6 on NWPU-Crowd. These results demonstrate the model’s strong robustness and generalization capability.

## 1. Introduction

Crowd counting has emerged as a prominent research focus in computer vision, aiming to accurately estimate the number of individuals in densely populated scenes. This capability plays a pivotal role in domains such as public safety [1] and smart city management [2]. For instance, in mass-transit hubs like airports and railway terminals, real-time crowd density estimation can deliver early warnings to prevent stampedes caused by overcrowding. From a broader urban perspective, these models generate high-resolution population-distribution maps, guiding resource allocation and informing smarter city planning and governance. However, crowd counting presents significant challenges, including substantial scale and density variations, severe occlusion, and complex backgrounds. To tackle these issues, diverse deep learning architectures have been developed, employing distinct strategies for multi-scale feature extraction and representation.

Deep learning models have substantially boosted crowd-counting accuracy; among them, Convolutional Neural Network (CNN) [3] and Transformer [4] architectures constitute the two dominant streams. Zhang et al. [5] proposed the Multi-column CNN (MCNN), which adapts to drastic scale variations caused by perspective or image resolution by equipping each column with filters of different receptive-field sizes; this design accepts images of arbitrary size, and the features learned by each column are inherently scale-adaptive. Li et al. [6] proposed a tandem extractor composed of a front-end standard CNN for 2D feature extraction and a back-end dilated CNN to enlarge the receptive field. Liu et al. [7] presented the Deep Structured Scale Integration (DSSI) Network that further alleviates scale variation through structured feature representation learning and hierarchically structured loss optimization. Nevertheless, CNNs rely on inductive biases such as locality and spatial invariance; although this yields fast inference, their limited receptive fields fail to capture long-range dependencies within the image.

Inspired by sequence modeling in natural language processing, researchers have designed the Transformer architecture, renowned for its long-range dependency modeling, into computer vision, achieving remarkable performance across diverse visual tasks. Vision Transformers (ViTs) leverage global self-attention to capture dependencies between arbitrary image patches, thereby overcoming the limited receptive fields of CNNs. However, the global attention mechanism incurs quadratic computational complexity, while modeling interactions between all patches often introduces redundancy in mainstream vision tasks. To mitigate computational overhead and attention redundancy, recent studies incorporate CNN-inspired inductive biases and adopt local self-attention within restricted receptive fields, as exemplified by EdgeViT [8] and RepViT [9]. Nevertheless, such locality-driven integrations inevitably compromise global modeling capacity, diminishing the ability to encode long-range dependencies. Consequently, designing hybrid architectures that effectively balance the computational efficiency of CNNs with the global representational power of Transformers remains a critical research frontier.

To address persistent challenges in crowd counting, we propose a Hybrid Multi-Scale Transformer-CNN U-shaped Net (HMSTUNet). Our model synergistically integrates the complementary strengths of ViTs and CNNs to achieve enhanced accuracy in crowd counting. Our principal contributions are threefold:

1. We propose a novel U-shaped encoder–decoder architecture, HMSTUNet. The encoder leverages the ConvNeXt backbone to extract hierarchical multi-level features from dense crowd images. The decoder incorporates a hybrid multi-scale network that effectively combines ViT and CNN components to capture both local and global features across different scales and levels. This U-shaped design ensures effective preservation and propagation of semantically critical features.

2. We design a new dual-branch fusion network that integrates transformer and CNN pathways to significantly boost feature representation capacity and counting accuracy. The dual-branch structure comprises a Multi-Scale Vision Transformer (MSViT) block, which employs a multi-scale multi-head attention mechanism, and a Dynamic Convolutional Attention Block (DCAB) that integrates a multi-dimensional attention mechanism with dynamic convolution operations. Furthermore, a multi-scale pyramid decoder (DecoderFPN) is developed to efficiently aggregate and refine multi-level features.

3. We demonstrate the effectiveness of HMSTUNet through extensive experiments on five widely used benchmarks. Our model achieves the best Mean Absolute Error (MAE) on all five datasets and the best Mean Square Error (MSE) on three of them, demonstrating a clear and comprehensive superiority. These results across diverse scenes underscore the robustness and strong generalization capability of our approach.

## 2. Related Work

### 2.1. Model Design for Crowd Counting

In computer vision, the predominant approach to crowd counting is density map regression. This method employs deep neural networks to predict a continuous density map from an input image, with the total count obtained by summing up the pixel values over the entire map. However, this paradigm faces three core challenges: (1) significant scale and density variations, where individuals in the foreground are larger and more sparse, while those in the background are smaller and densely packed; (2) pervasive occlusion and overlap, which often obscure the full-body visual cues of most individuals; and (3) considerable background clutter and variability due to varying illumination conditions and complex environments.

To mitigate these challenges, researchers have explored incorporating multi-scale feature fusion, attention mechanisms, and Transformer architectures to enhance model robustness and counting accuracy. Multi-scale feature fusion methods effectively mitigate scale variation by integrating features from different hierarchical levels. Liu et al. [10] proposed an end-to-end architecture that leverages a spatial pyramid to fuse multi-scale features, thereby adaptively encoding contextual information at appropriate scales. Han et al. [11] developed a selective inheritance learning method, which identifies optimal-scale features and employs a progressive strategy to inherit critical characteristics, demonstrating remarkable scale generalization. Attention mechanisms address imbalanced person density by enabling the model to focus on salient spatial regions or channels. Jiang et al. [12] designed a dual-branch network consisting of a density-aware attention network and an attention scaling network; the fusion of their outputs generates a refined density map, effectively mitigating uneven density distribution. Lin et al. [13] proposed a multifaceted attention network that integrates global and local attention, achieving superior performance. The self-attention mechanism inherent in Transformers excels at dynamically capturing global dependencies, providing distinct advantages for modeling complex scenes with scale variations, severe occlusion, and cluttered backgrounds. Tian et al. [14] employed a pyramid ViT as a backbone to capture global features, complemented by multi-scale dilated convolutions for density map prediction. Qian et al. [15] proposed a multi-scale U-Transformer network to capture multi-level semantic and fine-grained spatial features, leading to improved counting accuracy. Based on the aforementioned research, we have developed a U-shaped network architecture that incorporates a DCAB to extract multi-scale attention features and effectively integrates multi-level representations, thereby enhancing the overall model performance.

### 2.2. Vision Transformer

ViTs have demonstrated remarkable success across a range of visual tasks. The underlying mechanism involves applying self-attention to image patches and leveraging large-scale datasets together with data augmentation strategies [16], achieving outstanding performance in tasks such as image classification, object detection, and semantic segmentation. To handle large-scale variations among visual entities and high-resolution input images, the hierarchical Swin Transformer architecture with shifted windows was proposed [17]. It limits self-attention computation to local windows to reduce computational cost and employs a cross-window shifting mechanism to model relationships between patches across different windows. Within the inherently “columnar” topology of ViTs, incorporating multi-scale information has been shown to effectively improve visual task accuracy. As a result, several studies have focused on integrating multi-scale features. For instance, Conformer [18] integrates parallel self-attention and convolutional branches to capture multi-scale representations. MPViT [19] employs multi-scale patch embeddings and cascaded transformer blocks for multi-scale feature extraction, while CrossFormer [20] adopts cross-scale embedding layers combined with long-short distance attention mechanisms to enhance cross-granular dependency modeling. Inspired by these developments, we propose a multi-scale ViT block, termed MSViT, which significantly improves multi-scale feature extraction in dense crowd images and consequently boosts the overall network performance.

### 2.3. Loss Function

In response to the significant challenges posed by highly non-uniform spatial distributions and severe scale variations in crowd scenes, the development of specialized loss functions has become crucial for achieving accurate counting performance. Lempitsky et al. [21] established the density-map paradigm by reformulating counting as a per-pixel density estimation task. Building on this, Wan et al. [22] developed an adaptive density-map generator that dynamically integrates multi-scale blurred density maps, resulting in substantial improvements in map fidelity while maintaining computational efficiency in end-to-end training. Wang et al. [23] subsequently proposed DM-Count, a distribution matching framework that minimizes the Wasserstein distance between predicted and ground-truth density distributions to enforce spatial consistency. Building on the DM-Count approach, we formulated its corresponding loss function and conducted experimental evaluations, which demonstrated superior performance.

## 3. Methods

### 3.1. Overall Architecture

**Figure 1:**
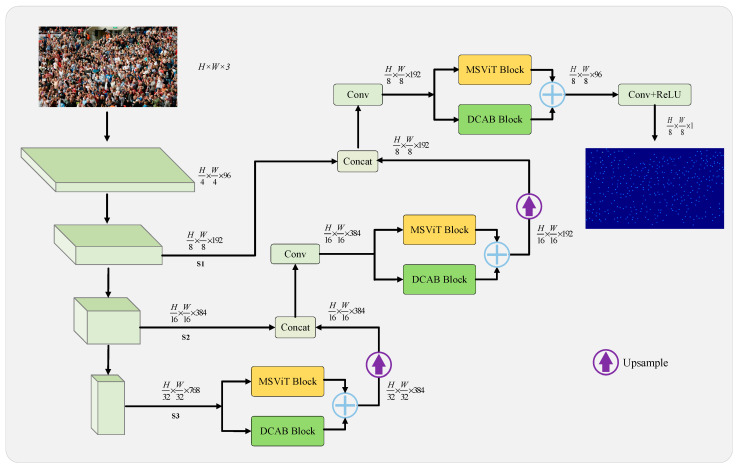
The overall architecture of the HMSTUNet.

Figure 1 illustrates the architectural design of HMSTUNet, which adopts an encoder–decoder U-Net structure tailored for crowd counting. The network is specifically designed to capture both high-level semantic information and low-level spatial details through enhanced skip connections. For the encoder, we employ ConvNeXt-Small, a state-of-the-art convolutional neural network recognized for its computational efficiency and superior performance in computer vision tasks. To handle the significant scale variations inherent in crowd scenes, we extract features from three distinct stages of ConvNeXt-Small. This multi-scale feature extraction strategy enables comprehensive representation learning across multiple hierarchical levels and resolutions, thereby enhancing the network’s capability for crowd counting. The decoder incorporates a sophisticated architectural innovation, consisting of two parallel blocks: a Multi-scale Vision Transformer (MSViT) block and a multi-dimensional feature extraction DCAB. This innovative network structure serves as the cornerstone for a multi-level feature fusion mechanism. Finally, the fused multi-level feature representations are transformed into a single-channel feature map, culminating in the generation of a density map that encapsulates both crowd count and spatial localization information. The proposed architecture represents a synergistic approach to crowd counting, integrating advanced neural network designs to address traditional challenges in complex, scale-variant crowd scenes.

### 3.2. Multi-Scale Vision Transformer Block

**Figure 2:**
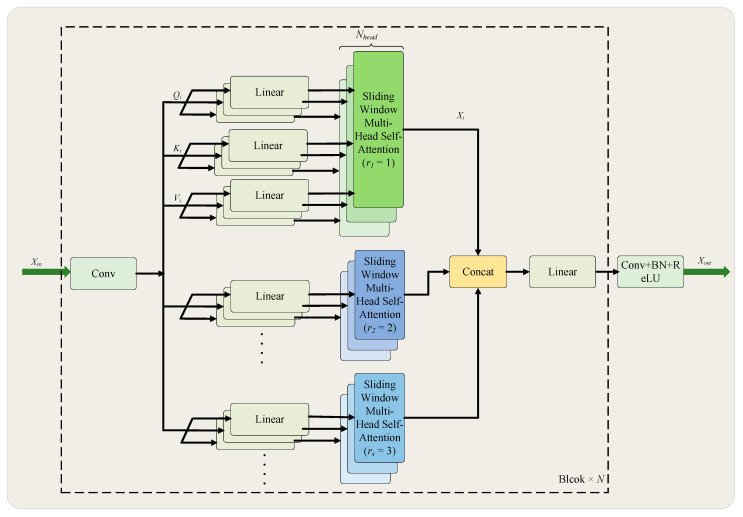
Illustration of MSViT Block.

To expand the receptive field while preserving spatial resolution, we propose the Multi-Scale Vision Transformer (MSViT) block. As illustrated in Figure 2, the block captures multi-scale contextual features by incorporating multiple dilation rates. The proposed architecture consists of N sequentially connected MSViT blocks, each containing s distinct dilation rates. By assigning different dilation rates to the self-attention heads, multi-scale features can be integrated within a single self-attention operation. This design not only facilitates effective interaction across scales but also significantly reduces computational redundancy in self-attention mechanisms, thereby lowering overall computational cost. Specifically, the input feature map is first projected from c channels to 3c channels via a 1 × 1 convolutional layer, generating Query (Q), Key (K), and Value (V) matrices. Subsequently, Sliding Window Multi-Head Self-Attention (SWMSA) is performed under s different dilation rates to enable multi-scale feature interaction. The resulting multi-scale features are then concatenated and fused. Finally, the channel-wise features at each spatial location are processed through a linear layer for transformation and fusion, followed by a 1 × 1 convolution that reduces the channel dimension by half to produce the output feature map. The calculation formulas for the MSViT block are as follows:

(1) $$Q_{i},K_{i},V_{i}={Conv}_{1\times 1}(X_{in})\;\;\;1\leq i\leq N_{head}$$

(2) $$X_{i}=SWMSA(Q_{i},K_{i},V_{i},r_{s})$$

(3) $$X_{out}=\sigma_{ReLU}(BN({Conv}_{1\times 1}(Linear(Concat[X_{1},X_{2}\cdots X_{N_{head}}]))))$$

In Equations (1) and (3), $X_{in}\in R^{H\times W\times C}$ denotes the input feature map; $X_{out}\in R^{H\times W\times \frac{C}{2}}$ denotes the output feature map; SWMSA(⋅) denotes the Sliding Window Multi-Head Self-Attention operation; $Q_{i}\in R^{H\times W\times C}$, $K_{i}\in R^{H\times W\times C}$ and $V_{i}\in R^{H\times W\times C}$ denote the query, key, and value feature matrices, respectively; $X_{i}\in R^{H\times W\times C}$ denotes the intermediate feature map; and $r_{s}$ denotes the set of dilation rates. In our implementation, the number of attention heads $N_{head}$ is set to 12, the dilation rates $r_{s}$ are [1, 2, 3], and the number of block repetitions N is 2. This specific combination of parameters is experimentally verified to deliver optimal model performance.

### 3.3. Dynamic Convolutional Attention Block

Inspired by the compelling effectiveness of dynamic convolution, we propose a novel Dynamic Convolutional Attention Block (DCAB) designed to operate in parallel with the MSViT block, thereby synergistically enhancing feature representation. The core innovation of our DCAB lies in its integrated multi-dimensional attention mechanism and parallel processing strategy, which dynamically learns complementary attention weights across four key dimensions of the convolution kernel: spatial size, number of kernels, input channels, and output channels. This design enables effective multi-scale feature extraction and significantly strengthens feature representation capabilities.

**Figure 3:**
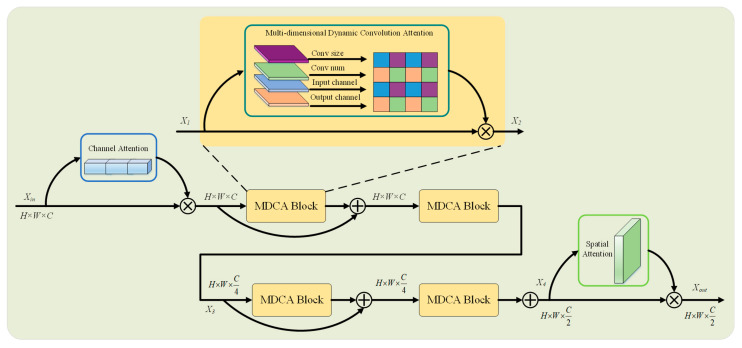
Illustration of DCAB.

As depicted in Figure 3, the input feature map $X_{in}$ first undergoes channel-wise attention enhancement. This step allows each channel to adaptively integrate global contextual information while aggregating features across all spatial positions. The resulting features are then processed by the Multi-dimensional Dynamic Convolution Attention (MDCA) block, which dynamically generates convolutional kernel weights conditioned on both the input data and the network’s learning state. This mechanism comprehensively captures feature interactions across the four kernel dimensions. By stacking multiple MDCA layers, the model can more effectively capture both dynamic and static feature variations in the scene, further improving its representational capacity. To optimize efficiency, a channel bottleneck mechanism is incorporated, where the number of channels is first reduced to one-quarter and subsequently restored to half of the original dimensionality, significantly lowering parameter count and computational cost. Residual connections are also employed to enhance generalization. Finally, a spatial attention block guides the model to focus on semantically salient regions in crowded images while suppressing irrelevant background noise, thereby enhancing robustness to occlusions and complex backgrounds. The computational flow of the proposed DCAB is defined by the following formulations:

(4) $$X_{1}=X_{in}⊙\sigma_{sigmoid}(\sigma_{ReLU}({Conv}_{1\times 1}(W\cdot AvgPool(X_{in}))))$$

(5) $$X_{3}=MDCA(MDCA(X_{1})+X_{1})$$

(6) $$X_{4}=MDCA(MDCA(X_{3})+X_{3})$$

(7) $$X_{out}=X_{4}⊙\sigma_{sigmoid}(Conv_{7\times 7}(Mean_{C}(X_{4})))$$

The core MDCA operation is implemented as:

(8) $$X_{2}=MDCA(X_{1})=X_{1}⊙\sum_{k=1}^{n}(\alpha_{sk}⊙\alpha_{nk}⊙\alpha_{ik}⊙\alpha_{ok}⊙W_{k})$$

Here, $X_{in}\in R^{H\times W\times C}$ and $X_{out}\in R^{H\times W\times \frac{C}{2}}$ denote the input and output feature maps, respectively; AvgPool denotes represent average pooling; W denotes the channel attention weight matrix; $Mean_{C}$ represents the mean operation along the channel dimension; $X_{1}\in R^{H\times W\times C}$, $X_{2}\in R^{H\times W\times C}$, $X_{3}\in R^{H\times W\times \frac{C}{4}}$ and $X_{4}\in R^{H\times W\times \frac{C}{2}}$ are intermediate feature maps; MDCA(⋅) refers to the Multi-Dimensional Convolutional Attention block; $\alpha_{sk}$, $\alpha_{nk}$, $\alpha_{ik}$ and $\alpha_{ok}$ denote the attention weights along the spatial size, number, input channel, and output channel dimensions, respectively; $W_{k}$ represents the weights of the k-th convolutional kernel.

### 3.4. Loss Function

Drawing inspiration from DM-Count, we propose a composite loss function that strategically combines three complementary components: a counting loss, an Optimal Transport (OT) loss, and a Variation (V) loss, as mathematically formulated in Equation (9):

(9) $$L=L_{C}+\lambda_{1}L_{OT}+\lambda_{2}L_{V}$$

where L represents the overall composite loss, $L_{C}$ denotes the counting loss, $L_{OT}$ corresponds to the optimal transport loss, and $L_{V}$ indicates the total variation loss. The hyperparameters $\lambda_{1}$ and $\lambda_{2}$, which control the relative contributions of the loss components, were both set to 0.1 in our experiments, a configuration that yielded the optimal performance.

The counting loss is designed to measure the absolute deviation between the predicted total count and the ground-truth total count, thereby enforcing global consistency in the overall cardinality estimation. It is formally defined as:

(10) $$L_{C}=\left|{\left\|z\right\|}_{1}-{\left\|\hat{z}\right\|}_{1}\right|$$

where $z\in R^{n}$ signifies the vectorized ground-truth density map, $\hat{z}\in R^{n}$ represents the vectorized predicted density map, and $\|\cdot {\|}_{1}$ denotes the L1 norm of a vector, representing the ummation of all pixel values in the density map.

The OT loss captures the discrepancy between the predicted density distribution and the ground-truth density distribution in the probability measure space. Specifically, we employ the Sinkhorn algorithm [24] to compute the optimal transport plan from the normalized predicted distribution to the normalized ground-truth distribution. The resulting optimal transport cost is then utilized as the loss value:

(11) $$L_{OT}=\left\langle \frac{\beta^{*}}{{\left\|\hat{z}\right\|}_{1}}-\frac{\left\langle \beta^{*},\hat{z}\right\rangle }{{\left\|\hat{z}\right\|}_{1}^{2}},\hat{z}\right\rangle$$

where $\beta^{*}$ denotes the optimal dual variable derived from the Sinkhorn iterations, and $\left\langle \cdot ,\cdot \right\rangle$ represents the vector inner product.

The Variation loss focuses on enhancing local structural similarity by comparing the intensity variations between adjacent pixels in the predicted and ground-truth density maps, thereby promoting spatial coherence. It is formulated as:

(12) $$L_{V}={\left\|z\right\|}_{1}\cdot {\left\|\frac{z}{{\left\|z\right\|}_{1}}-\frac{\hat{z}}{{\left\|\hat{z}\right\|}_{1}}\right\|}_{1}$$

## 4. Experiments

### 4.1. Datasets and Implementation Details

To comprehensively evaluate the performance of the proposed HMSTUNet, we conducted extensive experiments on five publicly available benchmark datasets and compared the results with state-of-the-art methods. The selected datasets—ShanghaiTech Part A and Part B [5], UCF_CC_50 [25], UCF-QNRF [26], and NWPU-Crowd [27]—vary considerably in scene complexity, crowd density distribution, and image resolution, as summarized in Table 1.

ShanghaiTech comprises 1198 annotated images with a total of 330,165 annotated instances, divided into two subsets: Part A (SHA) and Part B (SHB). SHA includes 482 images (300 for training and 182 for testing) collected from the Internet, while SHB consists of 716 images (400 for training and 316 for testing) obtained from real surveillance footage. Given its diverse scenes and highly imbalanced density distributions, this dataset represents a challenging benchmark in crowd counting.

UCF_CC_50 is a small yet highly variable dataset containing only 50 images with 63,075 annotated instances, where the number of people per image ranges from 94 to 4543. Encompassing various complex scenes and perspectives, this dataset presents significant challenges. Due to the limited data size, we followed the 5-fold cross-validation strategy recommended by the creators.

UCF-QNRF contains 1535 annotated images with a total of 1,251,642 annotated instances, split into 1201 training images and 334 test images. This dataset is characterized by dramatic variations in both crowd density and image resolution across diverse scenes, posing a rigorous test of model generalization capability.

NWPU-Crowd is currently the largest dataset, comprising 5109 images with 2,133,375 annotated instances. It is partitioned into training (3109 images), validation (500 images), and test (1500 images) sets. This dataset covers a wide spectrum of illumination conditions, viewing angles, and scene categories, and includes 351 negative samples (scenes without crowds). As the ground-truth annotations for the test set are not publicly released, we utilize the validation set for performance evaluation.

The encoder of HMSTUNet was initialized with the official ConvNeXt-Small model pretrained on ImageNet-1k. For data augmentation, we employed only random cropping and horizontal flipping. The crop sizes were set to 256 for SHA, 512 for SHB, UCF_CC_50, and UCF-QNRF, and 384 for NWPU-Crowd. All models were trained using the AdamW optimizer with an initial learning rate of 1 × 10^−5^. A batch size of 8 was used for the ShanghaiTech (SHA and SHB), UCF_CC_50, and NWPU-Crowd datasets, whereas a batch size of 32 was applied to the UCF-QNRF dataset. L2 regularization was incorporated with a coefficient of 0.005 for the ShanghaiTech datasets and 0.0001 for the UCF_CC_50, UCF-QNRF, and NWPU-Crowd datasets. All evaluations were performed on a single NVIDIA A100 GPU.

### 4.2. Evaluation Metrics

In crowd counting tasks, Mean Absolute Error (MAE) and Mean Square Error (MSE) are employed as standard evaluation metrics to quantify model performance. MAE measures the average absolute deviation between predicted counts and ground-truth annotations, providing an intuitive indicator of prediction accuracy. MSE, defined as the square root of the average squared errors, imposes a higher penalty on large discrepancies and thus effectively assesses model robustness against outliers and noisy samples. The mathematical formulations are expressed as:

(13) $$MAE=\frac{1}{N}\sum_{i=1}^{N}|y_{i}-{\hat{y}}_{i}|$$

(14) $$MSE=\sqrt{\frac{1}{N}\sum_{i=1}^{N}{\left|y_{i}-{\hat{y}}_{i}\right|}^{2}}$$

where N denotes the total number of test images, $y_{i}$ represents the ground-truth person count, and ${\hat{y}}_{i}$ signifies the predicted count for the i-th image.

### 4.3. Comparisons with State-of-the-Art Methods

In this section, we evaluate the performance of HMSTUNet by comparing it with 17 mainstream methods on five public crowd-counting datasets: SHA, SHB, UCF_CC_50, UCF-QNRF, and NWPU-Crowd. The comprehensive results are summarized in Table 2.

On the SHA dataset, our HMSTUNet achieved the best performance with an MAE of 49.1 and an MSE of 77.8, reducing the MAE by 0.2 and the MSE by 1.0 compared to the second-best method, PET [28]. For the SHB dataset, our model attained an MAE of 6.2 and an MSE of 10.3, matching the optimal MAE performance of PET and surpassing FGENet [29] and P2PNet [30] by 0.1 in MAE. In terms of MSE on SHB, HMSTUNet remained highly competitive, trailing the best result (PET) by only 0.5. On the UCF_CC_50 dataset, HMSTUNet achieved an MAE of 142.1 and an MSE of 192.7, reducing the MAE and MSE of the runner-up FGENet by 0.5 and 23.2, respectively, thus setting a new state-of-the-art. Similarly, on UCF-QNRF, our method achieved leading results with an MAE of 77.9 and an MSE of 132.5, surpassing the second-best method FGENet by 1.6 in MAE and 11.8 in MSE. On the NWPU-Crowd dataset, HMSTUNet attained an MAE of 43.2 and an MSE of 119.6, achieving the best MAE which is 8.2 lower than that of FIDTM [31], while its MSE ranked second, trailing FIDTM by 12.0.

Overall, HMSTUNet achieved the best MAE on all five datasets, demonstrating superior prediction accuracy. Regarding MSE, it attained the best performance on three datasets and remained highly competitive on the remaining two, evidencing robustness against noise and outliers.

**Table 2:** Performance comparison of different methods on SHA, SHB, UCF_CC_50, UCF-QNRF and NWPU-Crowd datasets. The best performance is in **boldface**, and the second best is underlined.

Method	Venue	Params(M)	SHA		SHB		UCF_CC_50		UCF-QNRF		NWPU-Crowd	
			MAE	MSE	MAE	MSE	MAE	MSE	MAE	MSE	MAE	MSE
MCNN [5]	CVPR’16	0.13	110.2	173.2	26.4	41.3	377.6	509.1	277.0	426.0	218.5	700.6
CSRNet [6]	CVPR’18	16.3	68.2	115.0	10.6	16.0	266.1	397.5	120.3	208.0	104.9	433.5
CANNet [10]	CVPR’19	18.1	62.3	100.0	7.8	12.2	212.2	243.7	107.0	183.0	93.6	489.9
SFCN+ [32]	CVPR’19	38.6	64.8	107.5	7.6	13.0	214.2	318.2	102.0	171.4	95.5	608.3
BL [33]	CVPR’19	21.5	62.8	101.8	7.7	12.7	229.3	308.2	88.7	154.8	93.6	470.4
AMRNet [34]	ECCV’20	59.3	61.6	98.4	7.0	11.0	184.0	265.8	86.6	152.2	-	-
DM-Count [23]	NeurIPS’20	21.5	59.7	95.7	7.4	11.8	211.0	291.5	85.6	148.3	88.4	388.6
LibraNet [35]	ECCV’20	17.9	55.9	97.1	7.3	11.3	181.2	262.2	88.1	143.7	-	-
Semi [36]	ICCV’21	16.7	66.9	125.6	12.3	17.9	-	-	130.3	226.3	105.8	445.3
P2PNet [30]	ICCV’21	19.2	52.7	85.1	6.3	9.9	172.7	256.2	85.3	154.5	77.4	362.0
CLTR [37]	ECCV’22	41.0	56.9	95.2	6.5	10.6	-	-	85.8	141.3	61.9	246.3
TransCrowd [38]	SCIS’22	90.4	66.1	105.1	9.3	16.1	-	-	97.2	168.5	88.4	400.5
FIDTM [31]	TMM’22	66.6	57.0	103.4	6.9	11.8	171.4	233.1	89.0	153.6	51.4	**107.6**
DMCNet [39]	WACV’23	-	58.5	84.6	8.6	13.7	-	-	-	-	96.5	164.0
PET [28]	ICCV’23	51.7	49.3	78.8	**6.2**	**9.7**	-	-	79.5	144.3	74.4	328.5
FGENet [29]	MMM’24	-	51.6	85.0	6.3	10.5	142.6	215.9	85.2	158.8	-	-
VMambaCC [40]	arXiv’24	-	51.9	81.3	7.5	12.5	-	-	-	-	88.4	144.7
MobileCount [41]	Neurocomputing	3.4	89.4	146.0	9.0	15.4	284.8	392.8	131.1	222.6	-	-
HMSTUNet (ours)	-	61.9	**49.1**	**77.8**	**6.2**	10.3	**142.1**	**192.7**	**77.9**	**132.5**	**43.2**	119.6

### 4.4. Ablation Study

#### 4.4.1. Component-Wise Ablation Study

As illustrated in Figure 1, the proposed HMSTUNet model is primarily composed of an encoder module and a decoder module. The encoder employs a pre-trained ConvNeXt-Small network as its backbone, while the decoder incorporates a hybrid feature extraction network combining MSViT and DCAB, which serves as the key component of our architectural design.

To validate the effectiveness of individual components, we conducted comprehensive ablation studies using a baseline model without the hybrid network on both the SHA and NWPU-Crowd datasets. The experimental results, summarized in Table 3, clearly demonstrate the contributions of each component:

1. The gradual incorporation of the combined MSViT and DCAB into successive decoder stages (S1 to S3) yields consistent and substantial performance gains across both datasets. For instance, on the SHA dataset, MAE decreases from 72.2 (Baseline) to 54.6 (Baseline + S3), confirming the effectiveness of local integration.

2. Isolating each component reveals their distinct strengths. The MSViT block excels at modeling global context and long-range dependencies, which is particularly beneficial for the large-scale and diverse NWPU-Crowd dataset, reducing MAE from 75.3 to 46.9. The DCAB focuses on adaptive local feature refinement, showing strong performance on both datasets.

3. The full integration of both MSViT and DCAB achieves the best results, outperforming all other configurations. This demonstrates their complementary nature: MSViT establishes a coherent global understanding, while DCAB performs precise local adjustment, a combination crucial for handling scale variation and congestion.

#### 4.4.2. Loss Function Ablation Study

As shown in Equation (9), the loss function comprises three terms: the counting loss ($L_{C}$), the optimal transport loss ($L_{OT}$), and the variation loss ($L_{V}$). By tuning the weight coefficients $\lambda_{1}$ and $\lambda_{2}$, we systematically evaluated the contribution of each loss component to model performance, with results summarized in Table 4. The experiments demonstrate that the model achieves optimal performance when both $\lambda_{1}$ and $\lambda_{2}$ are set to 0.1. This ablation analysis quantitatively validates the central role of counting loss in crowd counting tasks, while revealing that optimal transport loss enhances macro-level counting accuracy and distribution matching, and variation loss improves the fine-grained local fidelity and spatial smoothness of density maps.

### 4.5. Visualization

**Figure 4:**
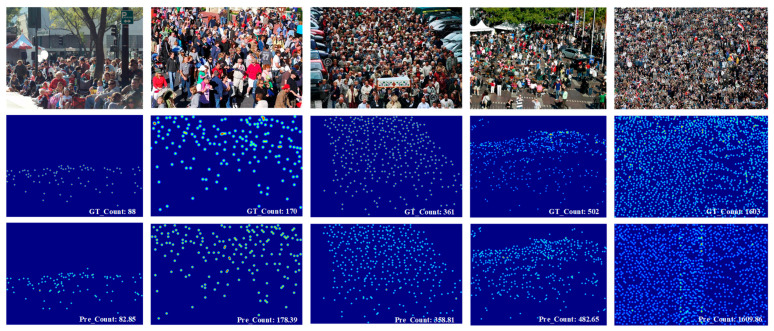
The visualization results on SHA datasets.

**Figure 5:**
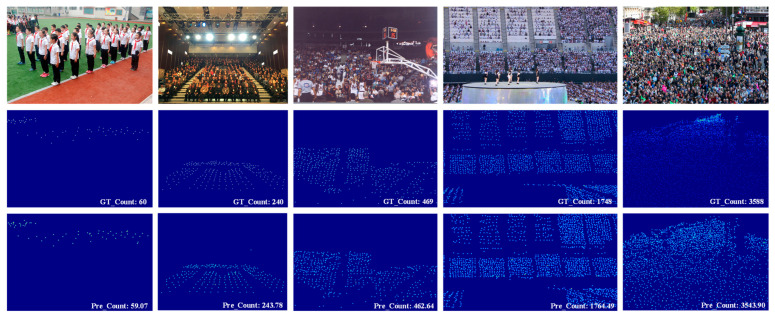
The visualization results on NWPU-Crowd datasets.

Figure 4 and Figure 5 depict the visualization results of the proposed HMSTUNet model on the SHA and NWPU-Crowd datasets, respectively. These results encompass a variety of complex scenarios, including diverse image scales, crowd densities, and illumination conditions, validating the model’s robustness across different challenging environments. The findings demonstrate that the proposed model is capable of generating high-quality density maps and maintaining accurate crowd counting performance in various complex scenes.

**Figure 6:**
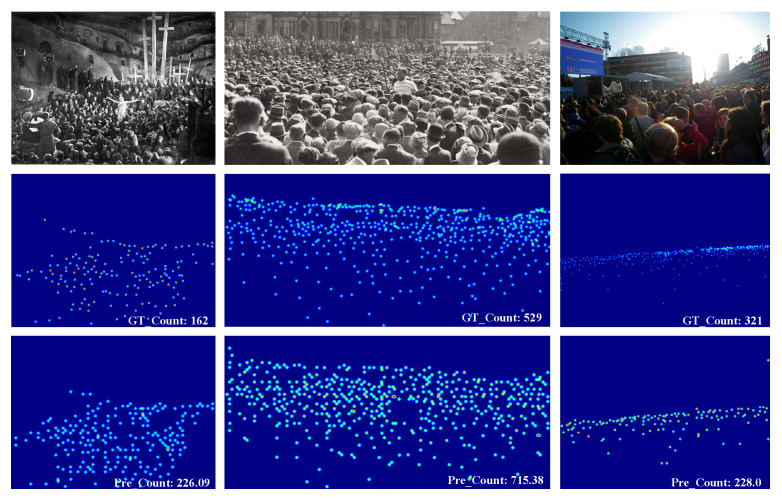
The visualization results under challenging conditions.

Figure 6 presents the visualization results of HMSTUNet under challenging conditions, such as low illumination and severe occlusion. The model exhibits degraded prediction performance under these conditions. Specifically, for the first sample set, the discrepancy between the predicted and ground-truth counts is 64.09, which remains within an acceptable range. In contrast, the prediction errors rise to 186.38 and 93 for the second and third sample sets, respectively, indicating that the model’s performance in such extreme cases still requires substantial improvement.

## 5. Conclusions

To address the challenges of severe occlusion, large scale variations, and non-uniform distribution in dense crowd counting, this paper proposes HMSTUNet, a hybrid multi-scale Transformer-CNN U-shaped encoder–decoder network. The model adopts a U-shaped architecture to extract multi-level image features, where the encoder is built upon a pre-trained ConvNeXt backbone and the decoder incorporates a hybrid multi-scale design combining Transformer and CNN components to effectively capture both local and global contextual information. Specifically, to address severe occlusion and overlapping in crowd images, we design a MSViT block for effectively modeling long-range dependencies. For handling significant scale changes and complex density patterns, we propose a DCAB that enhances the model’s capacity for capturing complex density patterns. Furthermore, a multi-scale pyramid DecoderFPN block is developed to aggregate feature information with larger receptive fields, while skip connections are incorporated to fuse deep semantic information with shallow spatial details.

Extensive experiments on five public crowd counting benchmarks demonstrate that HMSTUNet achieves state-of-the-art performance. It obtains the best MAE on all five datasets and the best MSE on three of them, indicating strong robustness and generalization ability. Although HMSTUNet provides an effective solution for dense crowd counting, its performance under challenging conditions such as poor illumination and extreme occlusion remains to be further improved. Future work will focus on two key directions to advance this line of research. First, we will conduct a deeper investigation into the multi-scale Vision Transformer and dynamic convolution frameworks to improve the model’s robustness and generalization in complex scenarios. Second, we will delve into the interpretability of the proposed architecture and extend the design principles of the Multi-Scale Transformer-CNN UNet to related tasks, such as person re-identification, to validate its transferability and broader applicability.

## Figures and Tables

**Table 1:** Statistics of crowd counting datasets.

Datasets	Number of Images	Training/Validation/Test	Count Statistics			Avg. Resolution
			Total	Max	Min	
SHA	482	300/-/182	241,677	3139	33	589 × 868
SHB	716	400/-/316	88,488	578	9	768 × 1024
UCF_CC_50	50	-	63,974	4543	94	2101 × 2888
UCF-QNRF	1535	1201/-/334	1,251,642	12,865	49	2013 × 2902
NWPU-Crowd	5109	3109/500/1500	2,133,375	20,033	0	2191 × 3209

**Table 3:** Component-wise ablation study on HMSTUNet. The best performance is in **boldface**.

Method	SHA		NWPU-Crowd	
	MAE	MSE	MAE	MSE
Baseline	72.2	129.3	80.3	245.2
Baseline + S1 (MSViT + DCAB)	58.4	90.7	57.9	182.4
Baseline + S2 (MSViT + DCAB)	56.2	87.9	55.6	180.7
Baseline + S3 (MSViT + DCAB)	54.6	86.8	53.4	178.3
Baseline + MSViT	52.1	83.6	46.9	138.4
Baseline + DCAB	51.8	83.4	45.1	137.5
Baseline + MSViT + DCAB	**49.1**	**77.8**	**43.2**	**119.6**

**Table 4:** Loss function ablation study. The best performance is in **boldface**.

Loss Function	SHA	
	MAE	MSE
$L_{C}$	56.2	95.9
$L_{C}+L_{OT}+L_{V}\;$	53.8	89.6
$L_{C}+\lambda_{1}L_{OT}+\lambda_{2}L_{V}\;(\lambda_{1}=0.1,\lambda_{2}=0.01)$	52.7	87.1
$L_{C}+\lambda_{1}L_{OT}+\lambda_{2}L_{V}\;(\lambda_{1}=0.1,\lambda_{2}=0.1)$	**49.1**	**77.8**

## Data Availability

The data that support the findings of this study are available from the corresponding author, lutianliang@ppsuc.edu.cn, upon reasonable request.
